# Too much or too little? The trajectory of systemic anti-cancer treatment throughout the last year of life of lung cancer patients in Norway

**DOI:** 10.1038/s44276-025-00183-w

**Published:** 2025-10-09

**Authors:** Steinar Solberg, Kathinka Schmidt Slørdahl, Marianne Aanerud, Odd Terje Brustugun, Bjørn Henning Grønberg, Nina Helbekkmo, Åslaug Helland, Yngvar Nilssen

**Affiliations:** 1 Department of Registration, Cancer Registry of Norway, Norwegian Institute of Public Health, Oslo, Norway; 2 Department of Cardiothoracic Surgery, Rikshospitalet, Oslo University Hospital, Oslo, Norway; 3 Department of Oncology, Oslo University Hospital, Oslo, Norway; 4 Department of Thoracic Medicine, Haukeland University Hospital, Bergen, Norway; 5 Department of Clinical Science, Faculty of Medicine, University of Bergen, Bergen, Norway; 6 Section of Oncology, Vestre Viken Hospital Trust, Drammen, Norway; 7 Institute of Clinical Medicine, University of Oslo, Oslo, Norway; 8 Department of Oncology, St Olavs Hospital, Trondheim University Hospital, Trondheim, Norway; 9 Department of Clinical and Molecular Medicine. NTNU, Norwegian University of Science and Technology, Trondheim, Norway; 10 Department of Pulmonology, University Hospital of North Norway, Tromsø, Norway

## Abstract

**Background:**

The use of systemic anti-cancer treatment (SACT) at the end of life (EOL) is controversial. The evidence and detailed description of the dynamics of its use are deficient, especially after the introduction of targeted therapies and immunotherapy.

**Methods:**

Clinical information about lung cancer patients dying in the years 2020–2023 was extracted from the Cancer Registry of Norway. Available data on intravenous and oral SACT enabled the calculation of the proportion of patients who received SACT each of the 360 days before death.

**Results:**

A total of 8953 patients were eligible for this study. At day 30, 7, and 1 before death, 8.9%, 1.3%, and 0.4% respectively, received SACT. The reduction was mainly caused by reduced use of chemotherapy and immunotherapy closer to death. Independent predictors for receiving SACT at day 30 before death were young age, male sex, small-cell lung cancer, short time from diagnosis to death, and good performance status.

**Conclusion:**

The presented low use of SACT at EOL has been achieved in a population where good survival has been documented. Patients with poor performance status and older age received less SACT than patients with good performance status and younger age.

## Introduction

Systemic anti-cancer treatment (SACT) needs to be used correctly to be effective and reduce the side effects to a minimum. Futile use of SACT at the end of life (EOL) wastes valuable time and puts patients at risk of unnecessary side effects. On the other hand, too little use of SACT may deprive the patient of relief of symptoms and prolongation of life. There are guidelines for initiating SACT [1] and for not initiating it at EOL [2], but criteria for terminating it are scarce. In addition, it is often difficult to predict treatment effect and prognosis for individual patients [3].

Optimal use of SACT is important for patients and their relatives and facilitates effective use of the health care system’s resources. To imply overtreatment, it has been stated that “chemotherapy use closer to the end of life is a marker of poor-quality care” [4], while others have shown that not all lung cancer patients receive appropriate treatment [5].

Studies have shown variation from 5% to 53% in the use of SACT during EOL [6–8]. Reasons for this disparity may be varying definitions of EOL, though most define it as the last month before death. Other reasons are variations in the health care system, the data included, and the study period. Importantly, there are few reports including data on modern, targeted therapies and immunotherapy.

Lung cancer is the cancer type causing most deaths and years of life lost [9]. The number of available targeted therapies has increased, and immunotherapy is now the standard treatment for tumours without oncogenic drivers [10]. The effect and tolerance of SACT have changed significantly since the chemotherapy era, and improved survival and symptom relief can be achieved in subgroups of patients in the advanced stage. The annual lung cancer report from the Norwegian Cancer Registry (CRN) showed that the survival (75^th^ percentile) for patients with metastatic adenocarcinomas increased from 14 to 32 months in the period 2013–2021 [11].

The Norwegian healthcare service is publicly funded and, in principle, equally available to all citizens, independent of age, sex, place of residence, and socioeconomic status. This also includes all cancer treatments. We aimed to assess the use of SACT in the last year of life for lung cancer patients using data from our national cancer registry.

## Materials and methods

### The Cancer Registry of Norway

The CRN was established by law in 1953, and reporting to the CRN is mandatory. The comparability, completeness, validity and timeliness of the registered data were estimated to be 99.3% for lung cancer in 2019–2023 [11, 12]. The CRN receives data from pathological and clinical examinations, radiotherapy departments, from the national patient registry and the national cause of death registry. From 2020 onwards, the CRN also receives data regarding SACT directly from the hospital systems [13] from three out of the four health regions. The Northern Norway region, comprising 10% of the Norwegian population, does not have a system for such electronic reporting. Additionally, the CRN receives information about all prescribed peroral cancer drugs. The CRN also runs the national quality register for lung cancer, which was established in 2013 and collects additional clinical data on diagnostics and surgery. After 2018, the completeness of these clinical diagnosis notifications has been over 90%, while it has been 100% for surgical notifications in the same period. More details have been described [14].

### Classification of variables

Information about stage (cTNM, version 8) and Eastern Cooperative Oncology Group Performance Status (ECOG PS) is received from the clinical notification from the time of diagnosis. Affiliation to the Health Region was based on the patients’ place of residence at the time of diagnosis.

### Systemic anti-cancer treatment

**Fig. 1:**
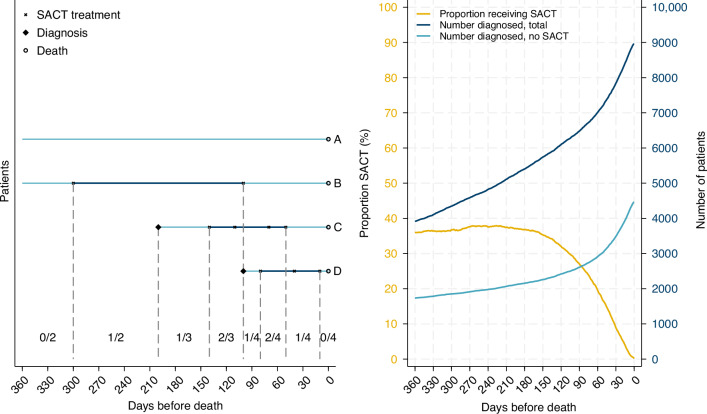
Lung cancer patients in Norway dying 2020–2023. The four cases in the left panel illustrate the calculations performed each day for all lung cancer patients included. Patient A and B were diagnosed at day 360 or earlier before death and both added one to the denominator until death at day zero. Patient A did not receive any SACT and did not add to the nominator, whereas patient B received SACT from day 300 to day 95 (illustrated by the bold part of the line) and accounted for one to the nominator each day in this period. Patient C and D were diagnosed on day 200 and 80 before death, respectively and added one to the denominator from these days. For the periods when they received SACT (bold parts of the lines), each of them added one to the nominator. The yellow curve in the right panel shows the proportion who received SACT, calculated as explained in the left panel. The dark blue curve shows the cumulative number of patients diagnosed per day before death, and the light blue shows the cumulative number diagnosed with lung cancer who did not receive any SACT.

SACT included both intravenous medications given at the hospitals and oral drugs prescribed to the patients. All medications given within eight days were classified as one treatment cycle. The treatment cycles were categorized as chemotherapy and immunotherapy, chemotherapy, immunotherapy or targeted therapy. To be comparable to other studies, day 30 before death was used for comparison of the use of SACT between different patient groups. As initial results showed the decline in the use of SACT started around 90 days before death, this point was used for comparison of the types of drugs used. We were able to calculate the number diagnosed (denominator) and the number treated (nominator) for each day throughout the last 360 days of life (Fig. 1).

### Statistical methods

The Pearson chi-squared test was used to assess differences between categories of the explanatory variables and a dichotomous variable indicating if a patient received SACT 30 days before death. A multivariable logistic regression was performed with the indicator of receiving SACT at day 30 before death (yes/no) as the dependent variable. This model was adjusted for time from diagnosis to death, age at diagnosis, sex, histology, ECOG PS, and Health Region. Every day during the last 360 days before death, we calculated the proportion of patients that were treated with SACT, overall and by age group, sex, stage, histology, health region, and ECOG PS. At every point (day) in time throughout the 360 days before death, we calculated the proportion of diagnosed patients that were treated with SACT. These calculations were done for every subgroup of age group, sex, stage, histology, regional health authority, symptoms, and ECOG PS. A sensitivity analysis including only patients who died from lung cancer was performed. A two-sided *p*-value < 0.05 was considered statistically significant. All analyses were performed using Stata 18.5 [15].

## Results

### The study population

Through 2020–2023, 11,333 patients registered with a diagnosis of lung cancer died in Norway. Of these, 2380 (21.0%) were excluded due to 1) the reported date of diagnosis being the same as date of death or later (*n* = 569, 5.0%), 2) diagnosed with lung cancer more than 10 years before death (*n* = 531, 4.7%), 3) primary sarcoma in the lung (*n* = 7, 0.1%), 4) unknown place of residence (*n* = 144, 1.3%), and 5) resident in the Northern Norway health region (*n* = 1129, 10.0%). Thus, the study group comprised 8953 patients. In patients diagnosed 10 years or more before death, and a diagnosis reported at the death certificate, 94 (17.7%) were reported with lung cancer. Thus, the majority in this group died from causes other than lung cancer, and this group was excluded.

The age as median and interquartile range of the study group was 75 (68–81) years at death, and 4163 (46.5%) were females (Table 1). A total of 3,911 (43.7%) patients had been diagnosed on day 360 before death or earlier, 5,404 (60.4%) on day 180, 7842 (87.6%) on day 30, and 1,111 (12.4%) were diagnosed less than 30 days before death.

**Table 1:** Lung cancer patients in Norway dying 2020–2023.

	Total	Diagnosed ≥ 30 days before death		Diagnosed < 30 days before death	
	*N* (%)	*N* (%)	%SACT	*N* (%)	%SACT
	8953 (100.0%)	7842 (100.0%)	8.9%	1111 (100.0%)	13.1%
**Time from diagnosis to death**					
<30 days	1111 (12.4%)			1111 (100.0%)	13.1%
30–90 days	1376 (15.4%)	1376 (17.5%)	8.9%		
91–180 days	1070 (12.0%)	1070 (13.6%)	15.6%		
181–360 days	1493 (16.7%)	1493 (19.0%)	11.1%		
360+ days	3903 (43.6%)	3903 (49.8%)	6.2%		
**Age at death**, years median (IQR)	73 (67–79)	73 (66–79)		76(70–81)	
**Age at death**, years median (IQR)	75 (68–81)	75 (68–80)		76(70–81)	
**Sex**					
Females	4163 (46.5%)	3684 (47.0%)	8.1%	479 (43.1%)	12.9%
Males	4790 (53.5%)	4158 (53.0%)	9.6%	632 (56.9%)	13.3%
**cTNM at diagnosis**					
I	1100 (12.3%)	1085 (13.8%)	3.1%	15 (1.4%)	0.0%
II	485 (5.4%)	477 (6.1%)	6.1%	8 (0.7%)	0.0%
III	1487 (16.6%)	1400 (17.9%)	8.9%	87 (7.8%)	21.8%
IV	4398 (49.1%)	3524 (44.9%)	12.5%	874 (78.7%)	13.4%
Unknown	1483 (16.6%)	1356 (17.3%)	5.2%	127 (11.4%)	7.9%
**Health region**					
South-Eastern Norway	5544 (61.9%)	4835 (61.7%)	8.7%	709 (63.8%)	11.0%
Western Norway	2000 (22.3%)	1784 (22.7%)	10.7%	216 (19.4%)	19.9%
Central Norway	1409 (15.7%)	1223 (15.6%)	7.0%	186 (16.7%)	13.4%
**Histology**					
Adenocarcinoma	3482 (45.2%)	3207 (46.4%)	10.2%	275 (34.6%)	8.4%
Squamous cell carcinoma	1747 (22.7%)	1627 (23.6%)	7.2%	120 (15.1%)	9.2%
NSCLC NOS	642 (8.3%)	556 (8.0%)	8.6%	86 (10.8%)	12.8%
Small cell lung cancer	1543 (20.0%)	1259 (18.2%)	14.0%	284 (35.7%)	33.1%
Other	288 (3.7%)	258 (3.7%)	8.5%	30 (3.8%)	20.0%
**ECOG PS at diagnosis**					
0	1402 (15.7%)	1379 (17.6%)	12.2%	23 (2.1%)	26.1%
1	2535 (28.3%)	2458 (31.3%)	10.9%	77 (6.9%)	33.8%
2	1704 (19.0%)	1535 (19.6%)	9.0%	169 (15.2%)	27.8%
3	1351 (15.1%)	918 (11.7%)	4.4%	433 (39.0%)	10.9%
4	444 (5.0%)	169 (2.2%)	1.2%	275 (24.8%)	1.8%
Unknown	1517 (16.9%)	1383 (17.6%)	6.0%	134 (12.1%)	11.2%

*ECOG PS* Eastern Cooperative Oncology Group Performance Status. *IQR* interquartile range. *NSCLC NOS* non-small cell lung cancer not otherwise specified.

Patient characteristics. The proportions in brackets add up to 100% by column for each group. The percentages without brackets show the proportions SACT received in the respective line.

The median time from diagnosis to start of SACT was 29 (15–77) days. The median time from the start of SACT until death was 270 (117–497) days. For those with small cell lung cancer (SCLC), the median time from diagnosis to treatment start was 12 (7–21) days, and the median time from treatment start to death 221 (103–381) days. A visualization of all SACT treatments given is shown in Supplementary Fig. S1.

### Overall

**Fig. 2:**
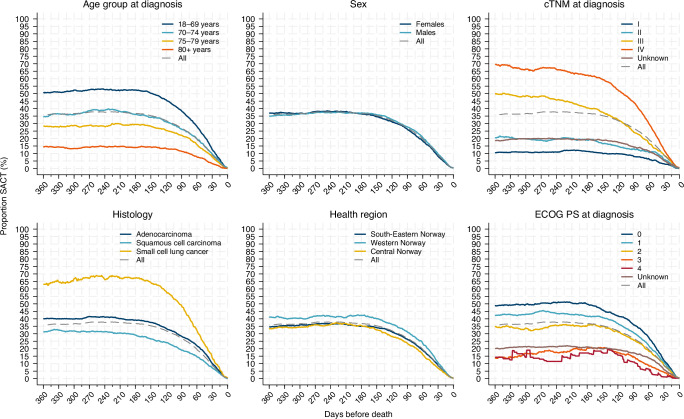
Lung cancer patients in Norway dying 2020–2023. The proportions given systemic anticancer treatment (SACT) at each day the last 360 days before death, by age group, sex, cTNM-stage at diagnosis, main histology groups, geography, and Eastern Cooperative Oncology Group Performance Status (ECOG PS) at diagnosis.

At day 360, 180, 90, 30, 7, and 1 before death, 35.9%, 36.8%, 27.1%, 8.9%, 1.3%, and 0.4% respectively, received SACT. The variation within the sub-groups is shown in Fig. 2.

**Fig. 3:**
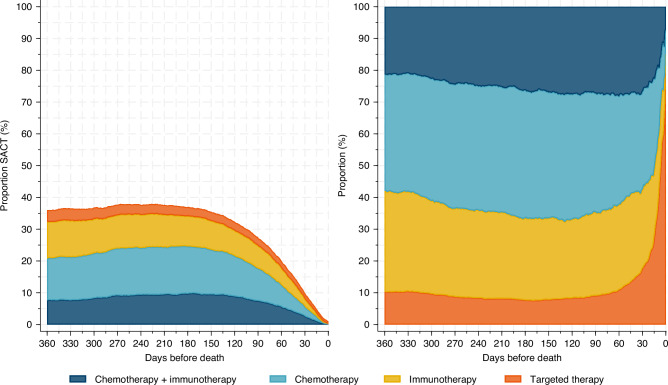
Lung cancer patients in Norway dying 2020–2023. The coloured areas show the proportions of the different drug groups of systemic anticancer therapy (SACT) given in the last 360 days before death. The left panel illustrates the proportions with all patients as denominators. In the right panel, the groups are stacked to illustrate the marked shift in drugs used the last few weeks of life.

Among those receiving SACT on day 90 before death, 27.3% received chemo- and immunotherapy, 37.3% received chemotherapy, 26.3% immunotherapy, and 9.1% targeted therapy. This distribution varied slightly until day 90, and on day 30, the corresponding proportions were 26.6%, 30.9%, 24.8%, and 17.7%. The increase in the proportion receiving targeted therapy started around day 90 and was 47.9% on day 7 before death (Fig. 3). On day 7 before death, 47.9% of the SACT given was targeted therapy. Further, 7.3% of the whole group received chemotherapy or immunotherapy on day 30 before death, and 0.7% on day 7.

The proportions of the types of SACT received, and the proportion of SACT being targeted therapy are shown in Fig. 3. The types of SACT given in the sub-groups are shown in supplementary Figs. S2–S7. Palliative radiotherapy had been given to 2608 (29.1%) patients, curative radiotherapy to 588 (6.6%), and 973 (10.9%) underwent surgery.

### Age

There were consistent differences in the proportions receiving SACT during the last year of life between the age groups (Fig. 2). At day 90 before death, 38.6% in the youngest group (18–69 years) and 11.5% in the oldest (80+ years) received SACT. On day 30, the corresponding figures were 12.6%, and 3.2% (*p* < 0.001).

### Sex

The trends for females and males received SACT intersected in the final 360 days of life (Fig. 2). Thirty days prior to death, 8.1% of females, and 9.6% of males were receiving SACT (*p* = 0.015). Ninety days before death, among those receiving SACT, 24.5% of females, and 27.2% of males received immunotherapy, while 12.5% of females and 6.2% of males received targeted therapy.

### Stage of disease

The differences in the proportions receiving SACT according to cTNM stage at diagnosis were consistent during the whole period (Fig. 2). On day 90, 44.5% in stage IV and 8.3% in stage I received SACT, and on day 30 the corresponding figures were 12.5% and 3.1%, respectively (Table 1) (*p* < 0.001).

### Histology

The differences between the use of SACT between the three main histologic subgroups were consistent during the last year of life (Fig. 2). After excluding patients with epidermal growth factor receptor- or anaplastic lymphoma kinase- positive adenocarcinoma, the changes in the curves were marginal (results not shown). On day 90 before death, 48.9%, 28.3%, and 19.1% of patients with small cell lung cancer, adenocarcinoma and squamous cell carcinoma (SCC), respectively, received SACT. On day 30, the corresponding figures were 14.0%, 10.2%, and 7.2% respectively (*p* = 0.001). In patients diagnosed less than 30 days before death, the proportion receiving SACT was highest for small cell lung cancer (33.1%) (Table 1).

Among those receiving SACT at day 90 before death, targeted therapy was given to 18.5% of the patients with AC and to almost none with SCC and SCLC. On this day, chemotherapy was given to 17.7% with AC, 30.8% with SCC and to 71.4% with SCLC, and immunotherapy to 24.7% with AC, 57.1% with SCC, and 17.6% with SCLC.

### Health Regions

A higher proportion of the patients in Western Norway received SACT than in the other two regions (Fig. 2) and on day 90 before death, 31.2% of those in Western Norway, 23.6% in Central Norway, and 26.3% in South-Eastern Norway received SACT. On day 30, the corresponding figures were 10.7% and 8.7%, respectively (*p* = 0.022).

### Performance Status (ECOG PS)

There were differences in the percentage of patients receiving SACT by ECOG PS at diagnosis (Fig. 2). At day 90 before death, 36.2% of patients with ECOG PS 0 received SACT, while the percentage was 11.1% among those with ECOG PS 4. At day 30, the proportions were 12.2% and 1.2%, respectively (*p* = 0.006).

### Time from diagnosis to death

**Fig. 4:**
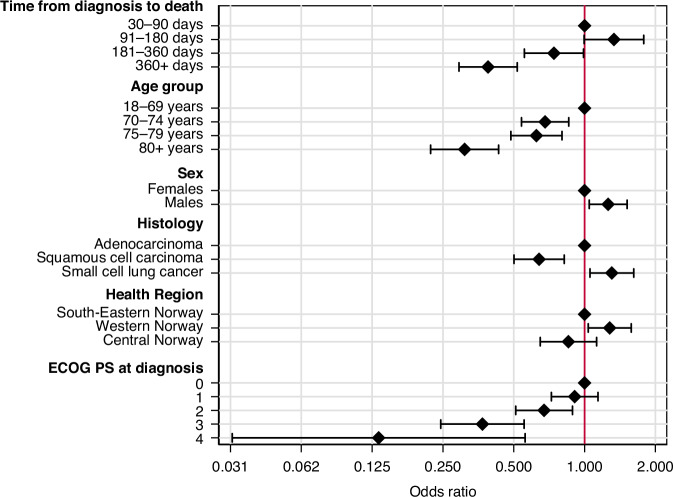
Lung cancer patients in Norway dying 2020–2023. A Forest plot showing the predictors for receiving systemic anticancer treatment at day 30 before death. The exact odds ratios and 95% confidence intervals are listed in Table 2.

Of patients diagnosed less than 30 days before death, 13.1% received SACT (Table 1). Longer time from diagnosis to death was an independent predictor for not receiving SACT (*p* < 0.001) (Fig. 4 and Table 2).

**Table 2:** Lung cancer patients in Norway dying 2020–2023. Multivariable regression analysis of predictors for being allocated to systemic anticancer treatment (SACT) at day 30 before death.

	OR	95% CI	*p*-value
**Time from diagnosis to death**			
30–90 days	1.00		
91–180 days	1.33	1.00–1.79	0.053
181–360 days	0.74	0.55–0.99	0.042
360+ days	0.39	0.29–0.52	<0.001
**Age group at diagnosis**			
18–69 years	1.00		
70–74 years	0.68	0.54–0.86	0.001
75–79 years	0.62	0.49–0.80	<0.001
80+ years	0.31	0.22–0.43	<0.001
**Sex**			
Females	1.00		
Males	1.26	1.05–1.52	0.015
**Histology**			
Adenocarcinoma	1.00		
Squamous cell carcinoma	0.64	0.50–0.82	<0.001
Small cell lung cancer	1.31	1.05–1.62	0.015
**Health Region**			
South-Eastern Norway	1.00		
Western Norway	1.28	1.04–1.58	0.022
Central Norway	0.85	0.65–1.12	0.261
**ECOG PS at diagnosis**			
0	1.00		
1	0.91	0.72–1.14	0.406
2	0.67	0.51–0.89	0.005
3	0.37	0.24–0.55	<0.001
4	0.13	0.03–0.56	0.006

*CI* confidence interval, *ECOG* PS Eastern Cooperative Oncology Group Performance Status, *OR* Odds ratio.

Among those diagnosed less than 30 days before death, the percentage receiving SACT varied from 1.8% of patients with ECOG PS 4 to 33.1% of patients with SCLC (Table 1).

## Discussion

The present study showed a marked reduction in the proportion of lung cancer patients receiving SACT in the last few months of life. At day 30, 7, and 1 before death, 8.9%, 1.7%, and 0.4%, respectively, received SACT. Predictors for receiving SACT at day 30 before death were being diagnosed with lung cancer 30–90 days before death, young age, male sex, small cell lung cancer, living in Western Norway, and good performance status at diagnosis. Being diagnosed more than 90 days before death and having SCC were predictors of not receiving SACT. Among those who received SACT, the relative proportion receiving targeted therapy increased during the last days of life.

The high proportion (5.0%) diagnosed at death or later may be explained by that this study has a date of death, and not diagnosis at the start point. In patients diagnosed with lung cancer in Norway, 2020–2024, 2.6% were diagnosed at autopsy. A sensitivity analysis including only patients dying from lung cancer revealed some changes in the level of significance, but no change in the main findings or conclusions.

The results from the present study showed that the use of SACT at EOL was at a low level compared to other studies [6–8]. In a nationwide French study, 23.6% of the lung cancer patients received chemotherapy, including targeted therapy the last month of life [6]. In a recent single-centre Austrian study, 42.6% of the lung cancer patients received SACT in the last month of life [16]. The use of SACT in lung cancer patients seemed to be higher than in other tumour types [6, 16].

A decreased use of SACT with increasing age might be reasonable and expected. However, it is unclear if the marked reduction by increasing age has been based on sound criteria and in agreement with the patients, or based on exaggerated assumptions of reduced tolerance, high risk, or reduced effect. Rostoft et al. found that the oldest cancer patients were less often hospitalized, less often given chemotherapy, and received less specialized palliative care during the last year of life [17, 18], which may indicate an undertreatment. In the present material, the use of SACT in the youngest age group was three times higher than in the oldest throughout most of the last year of life. An age gradient is in line with other findings [6, 19] and may be explained by increased emotional distress [20] and less comorbidity in the young. Further, the young patients seem to have an increased willingness to accept toxicity and meager chance of cure after SACT [21].

After the advent of the new drug types, there has been a shift from chemotherapy to more use of immunotherapy in the treatment of lung cancer [10, 22]. The present finding of a marked increase in proportion given targeted therapy, and a corresponding reduction of chemotherapy and immunotherapy closer to death, is according to guidelines advocating avoidance of chemotherapy and immunotherapy in the last few weeks of the patient’s lives [23].

Our findings in those receiving SACT of increased use of immunotherapy and targeted therapy, and a corresponding reduction of chemotherapy by increasing age, is in line with the guidelines [24]. The reduced use of immunotherapy in the oldest may be based on the finding of reduced survival benefit in the old [25]. Also, the benefit of immunotherapy on survival seems less favourable in register data than in controlled clinical studies [10].

Except for adjuvant chemotherapy after surgery, SACT is not indicated for patients in cTNM stage I and II at diagnosis. A proportion of these have, however, received SACT, indicating a long time since diagnosis and that the disease has probably relapsed after radical treatment by surgery or stereotactic radiation. Whether all patients with relapse have been identified and treated correctly cannot be answered by the present data.

A high proportion of patients with SCLC received SACT during the last year of life, and most of the SACT given was chemotherapy. A high proportion of patients diagnosed with SCLC less than 30 days before death received SACT. This may be justified by a higher response rate and positive outcomes seen in sub-groups of SCLC patients [11, 14].

The present finding of differences in the use of SACT between the health regions might indicate systematic geographical differences in the resources used for the treatment of lung cancer patients. However, a recent Norwegian study presented variation between the health regions in treatment and survival for small-cell lung cancer, and here, the ranking of which regions treated the most patients was quite different from the present findings [14]. Thus, differences in treatment culture seem more likely as an explanation than differences within the patient groups or available hospital resources.

Our finding of decreased use of SACT by reduced performance status is in line with findings regarding curative treatment after diagnosis [5], at EOL [16], and is also according to guidelines [23, 24].

This study has some limitations. The data on stage and performance status are from the time of diagnosis. Thus, both may have changed at the time of treatment from when we do not have these data. Any conclusion based on these data must be cautious. In addition, data on socioeconomic status, co-morbidity, and smoking were unavailable. Despite universal and public healthcare, the care of cancer patients in Norway is influenced by socioeconomic status [17]. As the Northern health region was unable to report the use of SACT, we miss 10% of the population from being completely national. Despite these limitations, this study has some strengths as well. This study continuously reports SACT throughout the last year of life for all patients in a national cohort over four years, and we have included all SACT given in the last year of life. As these vary from day to day, it was considered that the proportion given SACT each day was more relevant than giving the proportion for a time interval.

Our data does not support the notion of a general overtreatment with cancer drugs at the end of life for lung cancer patients in Norway. Thus, the change away from nihilism [26] does not seem to have been replaced by overtreatment. This low use of SACT could raise suspicion of undertreatment. Though a study comprising 11 European countries shows that Norway met the benchmark for the use of SACT in advanced non-small cell lung cancer from 2019 [24]. Further, Norway has the best survival outcome among the Nordic countries [27], and a median and relative survival of 18 months and 31.7% [11], respectively, which indicates that there has been no undertreatment.

As both overtreatment and undertreatment seem unlikely, the present findings may be used as a reference for treatment with cancer drugs during the last period for lung cancer patients. However, attention should be directed towards the SACT use in the elderly and those with a long time since diagnosis to ensure that the appropriate patients are receiving correct treatment.

## Conclusion

The presented low use of SACT at EOL has been achieved in a population where good survival has been documented. Patients with poor performance status and older age received less SACT than patients with good performance status and younger age.

## Supplementary information

Supplementary materials

## Data Availability

Data underlying this article can be requested from the Cancer Registry of Norway through Data underlying this article can be requested from the Cancer Registry of Norway through https://helsedata.no/en/.

## References

[1] Saini KS, Twelves C. Determining lines of therapy in patients with solid cancers: a proposed new systematic and comprehensive framework. Br J Cancer. 2021;125:155–63. 33850304 10.1038/s41416-021-01319-8 PMC8292475

[2] Schnipper LE, Smith TJ, Raghavan D, Blayney DW, Ganz PA, Mulvey TM, et al. American Society of Clinical Oncology identifies five key opportunities to improve care and reduce costs: the top five list for oncology. J Clin Oncol: J Am Soc Clin Oncol. 2012;30:1715–24. 10.1200/JCO.2012.42.8375 22493340

[3] Hui D, Park M, Liu D, Paiva CE, Suh SY, Morita T, et al. Clinician prediction of survival versus the Palliative Prognostic Score: Which approach is more accurate?. Eur J Cancer. 2016;64:89–95. 27372208 10.1016/j.ejca.2016.05.009 PMC4969216

[4] Woldie I, Elfiki T, Kulkarni S, Springer C, McArthur E, Freeman N. Chemotherapy during the last 30 days of life and the role of palliative care referral, a single center experience. BMC Palliat Care. 2022;21:20. 35125092 10.1186/s12904-022-00910-x PMC8819957

[5] Langballe R, Jakobsen E, Iachina M, Karlsen RV, Ehlers JH, Svendsen MN, et al. Who are the vulnerable lung cancer patients at risk for not receiving first-line curative or palliative treatment?. Acta oncol. 2023;62:1301–8. 37656828 10.1080/0284186X.2023.2252581

[6] Rochigneux P, Raoul JL, Beaussant Y, Aubry R, Goldwasser F, Tournigand C, et al. Use of chemotherapy near the end of life: what factors matter?. Ann Oncol. 2017;28:809–17. 27993817 10.1093/annonc/mdw654

[7] Bekelman JE, Halpern SD, Blankart CR, Bynum JP, Cohen J, Fowler R, et al. Comparison of site of death, health care utilization, and hospital expenditures for patients dying with cancer in 7 developed countries. JAMA. 2016;315:272–83. 26784775 10.1001/jama.2015.18603

[8] Kao S, Shafiq J, Vardy J, Adams D. Use of chemotherapy at end of life in oncology patients. Ann Oncol. 2009;20:1555–9. 19468033 10.1093/annonc/mdp027

[9] Brustugun OT, Moller B, Helland A. Years of life lost as a measure of cancer burden on a national level. Br J Cancer. 2014;111:1014–20. 24983370 10.1038/bjc.2014.364 PMC4150272

[10] Hektoen HH, Tsuruda KM, Fjellbirkeland L, Nilssen Y, Brustugun OT, Andreassen BK. Real-world evidence for pembrolizumab in non-small cell lung cancer: a nationwide cohort study. Br J Cancer. 2024. 10.1038/s41416-024-02895-1 PMC11724112 39489879

[11] The Cancer Registry of Norway. Annual report. National quality register for Lung Cancer 2023 2024 [Available from: https://www.kreftregisteret.no/Generelt/Rapporter/Arsrapport-fra-kvalitetsregistrene/Arsrapport-for-lungekreft/arsrapport-for-lungekreft-2023/.

[12] Larsen IK, Småstuen M, Johannesen TB, Langmark F, Parkin DM, Bray F, et al. Data quality at the Cancer Registry of Norway: an overview of comparability, completeness, validity and timeliness. Eur J Cancer. 2009;45:1218–31. 19091545 10.1016/j.ejca.2008.10.037

[13] The Cancer Registry of Norway. INSPIRE - information about drug cancer treatment. Lung cancer.2024 01.10.2024. Available from: https://www.kreftregisteret.no/Forskning/Prosjekter/inspire/inspirelungekreft/.

[14] Nilssen Y, Brustugun OT, Fjellbirkeland L, Grønberg BH, Haram PM, Helbekkmo N, et al. Small Cell Lung Cancer in Norway: Patterns of Care by Health Region and Survival Trends. Clin Lung cancer. 2024;25:e221–e8.e3. 38692990 10.1016/j.cllc.2024.04.002

[15] StataCorp. 2023. Stata Statistical Software: Release 18. College Station, TX: StataCorp LLC. 2023.

[16] Le NS, Zeybek A, Hackner K, Gottsauner-Wolf S, Groissenberger I, Jutz F, et al. Systemic anticancer therapy near the end of life: an analysis of factors influencing treatment in advanced tumor disease. ESMO Open. 2024;9:103683. 39214050 10.1016/j.esmoop.2024.103683 PMC11402042

[17] Rostoft S, Thomas MJ, Slaaen M, Møller B, Nesbakken A, Syse A. Hospital use and cancer treatment by age and socioeconomic status in the last year of life: A Norwegian population-based study of patients dying of cancer. J Geriatr Oncol. 2024;15:101683. 38065011 10.1016/j.jgo.2023.101683

[18] Rostoft S, Thomas MJ, Slaaen M, Møller B, Syse A. The effect of age on specialized palliative care use in the last year of life for patients who die of cancer: A nationwide study from Norway. J Geriatr Oncol. 2022;13:1103–10. 35973916 10.1016/j.jgo.2022.08.002

[19] Bähler C, Näpflin M, Scherer M, Blozik E. Continuity of care and treatment intensity at the end of life in Swiss cancer patients. Eur J Public Health. 2023;33:396–402. 37029913 10.1093/eurpub/ckad047 PMC10234661

[20] Kirkova J, Walsh D, Rybicki L, Davis MP, Aktas A, Tao J, et al. Symptom severity and distress in advanced cancer. Palliat Med. 2010;24:330–9. 20015920 10.1177/0269216309356380

[21] Bremnes RM, Andersen K, Wist EA. Cancer patients, doctors and nurses vary in their willingness to undertake cancer chemotherapy. Eur J Cancer. 1995;31a:1955–9. 8562147 10.1016/0959-8049(95)00513-7

[22] Kerekes DM, Frey AE, Prsic EH, Tran TT, Clune JE, Sznol M, et al. Immunotherapy initiation at the end of life in patients with metastatic cancer in the US. JAMA Oncol. 2024;10:342–51. 10.1001/jamaoncol.2023.6025 PMC10767643 38175659

[23] Geyer T, Le NS, Groissenberger I, Jutz F, Tschurlovich L, Kreye G. Systemic anticancer treatment near the end of life: a narrative literature review. Curr Treat Options Oncol. 2023;24:1328–50. 37501037 10.1007/s11864-023-01115-x PMC10547806

[24] Hofmarcher T, Lindgren P, Wilking N. Systemic anti-cancer therapy patterns in advanced non-small cell lung cancer in Europe. J Cancer Policy. 2022;34:100362. 36087918 10.1016/j.jcpo.2022.100362

[25] Voruganti T, Soulos PR, Mamtani R, Presley CJ, Gross CP. Association between age and survival trends in advanced non-small cell lung cancer after adoption of immunotherapy. JAMA Oncol. 2023;9:334–41. 36701150 10.1001/jamaoncol.2022.6901 PMC9880865

[26] Nilssen Y, Strand TE, Fjellbirkeland L, Bartnes K, Møller B. Lung cancer survival in Norway, 1997-2011: from nihilism to optimism. Eur Respir J. 2016;47:275–87. 26541525 10.1183/13993003.00650-2015

[27] Tichanek F, Försti A, Hemminki O, Hemminki A, Hemminki K. Survival in lung cancer in the nordic countries through a half century. Clin Epidemiol. 2023;15:503–10. 37153073 10.2147/CLEP.S406606 PMC10162394

