# Supplementary material for: Too much or too little? The trajectory of systemic anti-cancer treatment throughout the last year of life of lung cancer patients in Norway
Source: BJC Rep. 2025 Oct 9;3:70. doi: 10.1038/s44276-025-00183-w (PMC12511628; doi:10.1038/s44276-025-00183-w)
Supplement: Supplementary file 1 — Supplementary materials [file 44276_2025_183_MOESM1_ESM.docx]

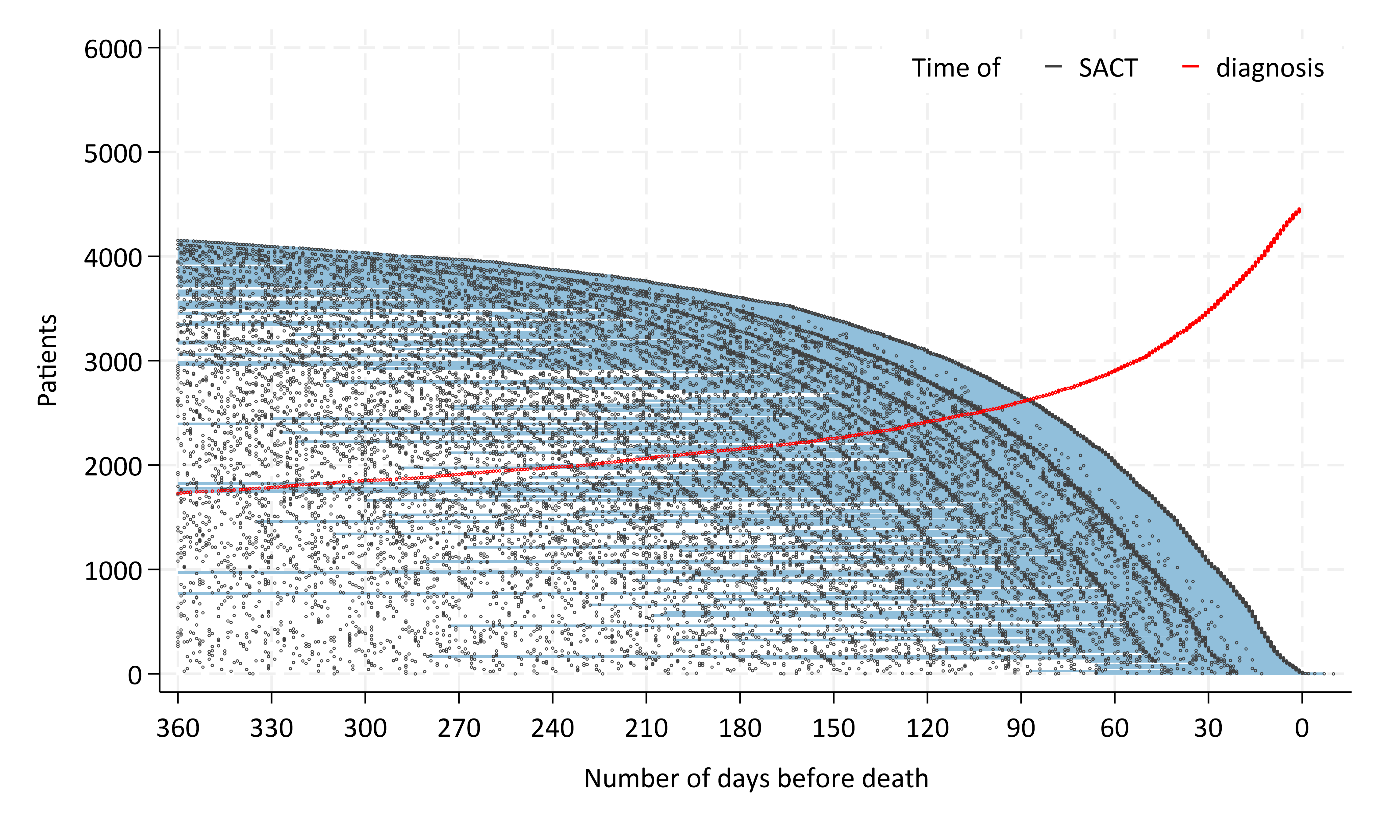
**Figure S1.** Lung cancer patients in Norway dying 2020-2023. A scatterplot visualizing all SACT given in the last year of life. Every cycle of each patient's SACTs are indicated by a black point, and for patients who did not receive SACT, the time from diagnosis to death is indicated by a red point. The blue lines indicate the start and end of a patient's final SACT cycle. A treatment cycle was assumed to be 25 days.


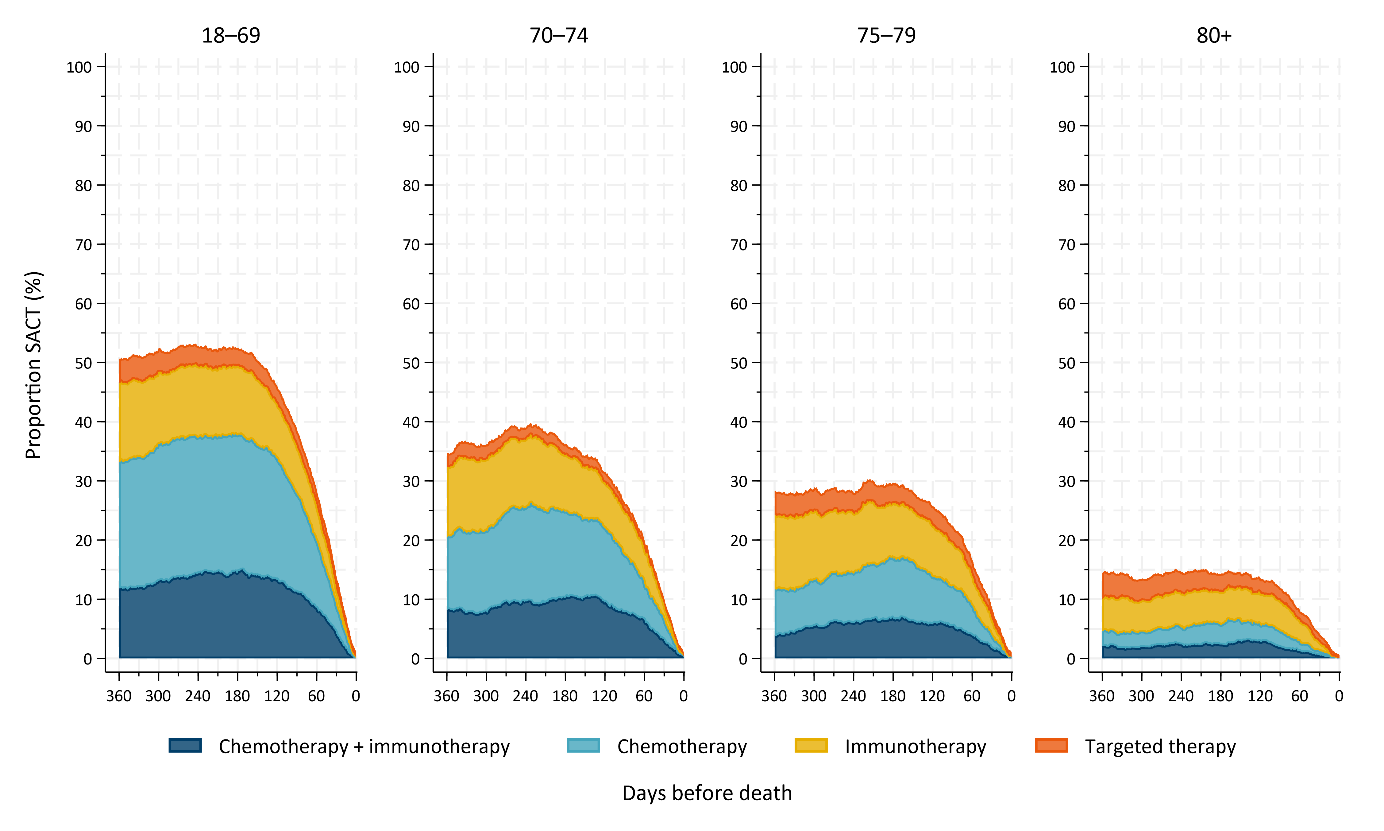
**Figure S2**. Lung cancer patients in Norway dying 2020-2023. The proportions of types of SACT by age group.


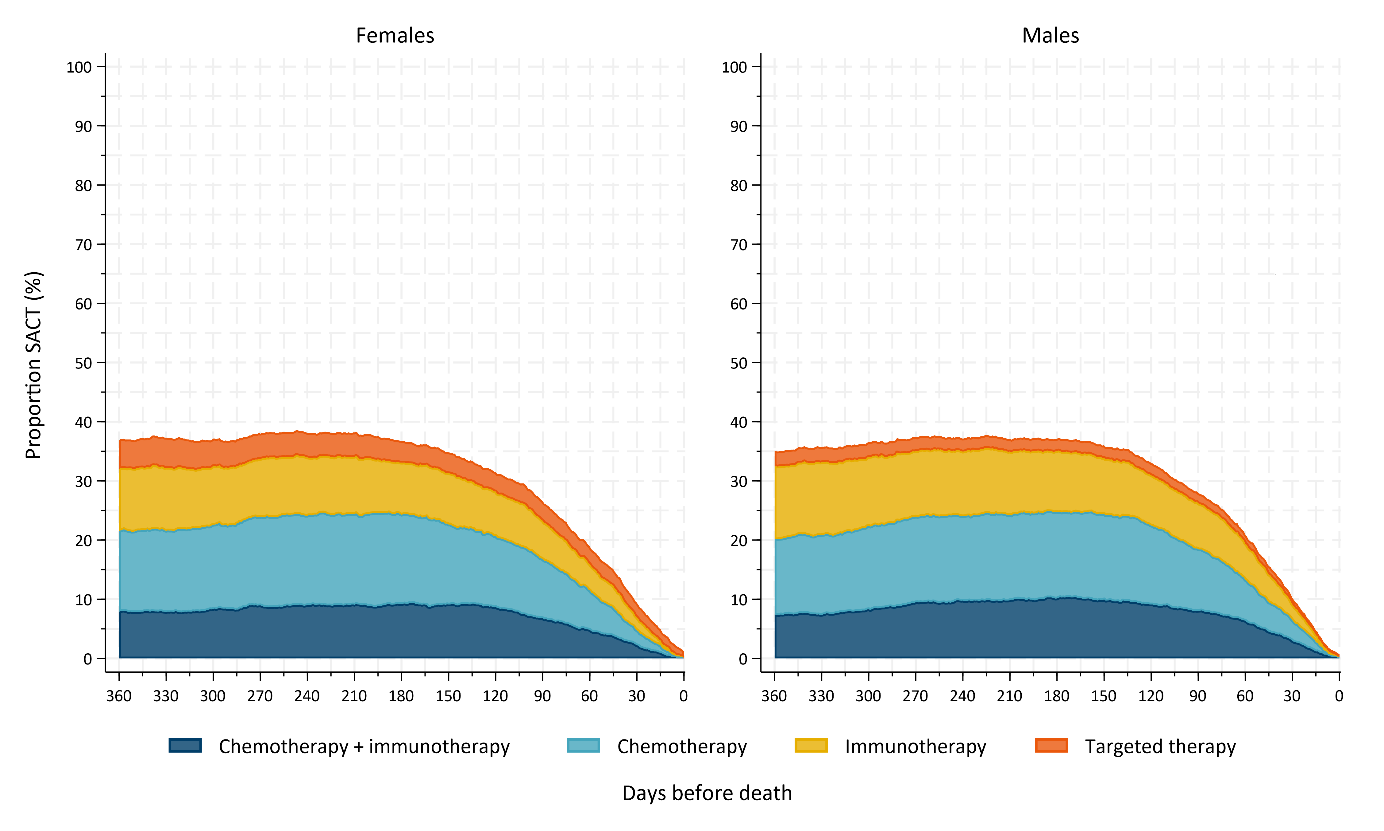
**Figure S3.** Lung cancer patients in Norway dying 2020-2023. The proportions of types of SACT by sex.


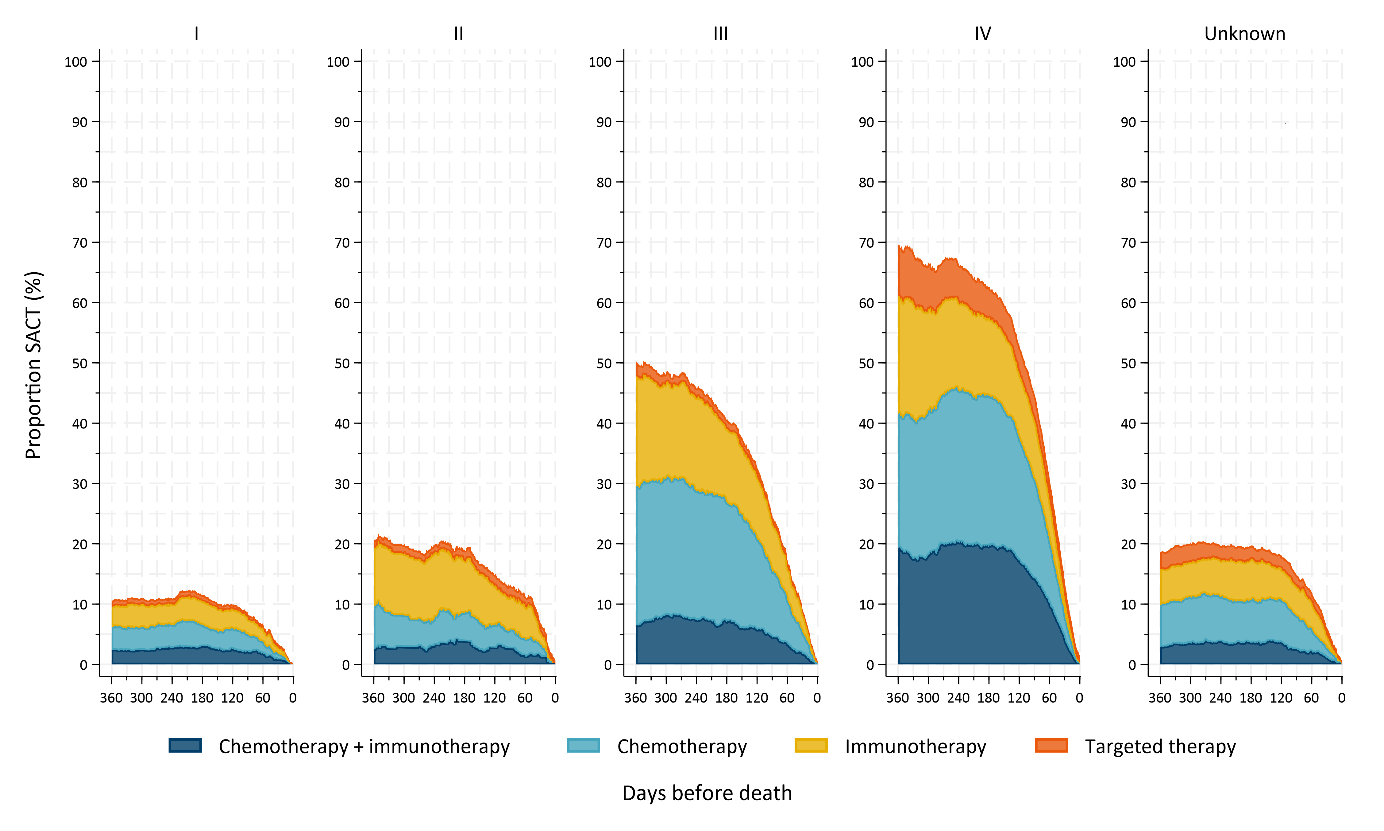
**Figure S4.** Lung cancer patients in Norway dying 2020-2023. The proportions of types of SACT by cTNM-stage.


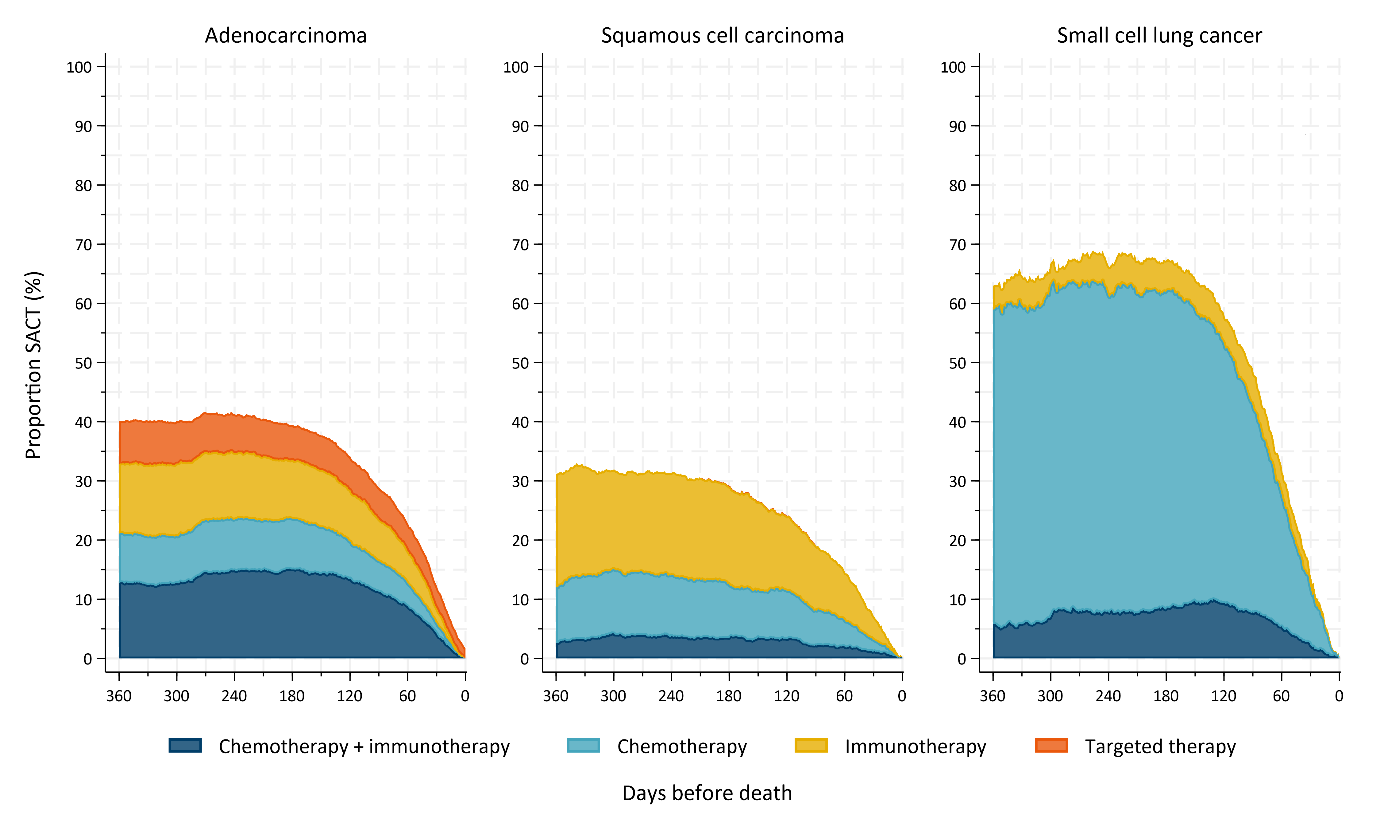
**Figure S5.** Lung cancer patients in Norway dying 2020-2023. The proportions of types of SACT by histology.


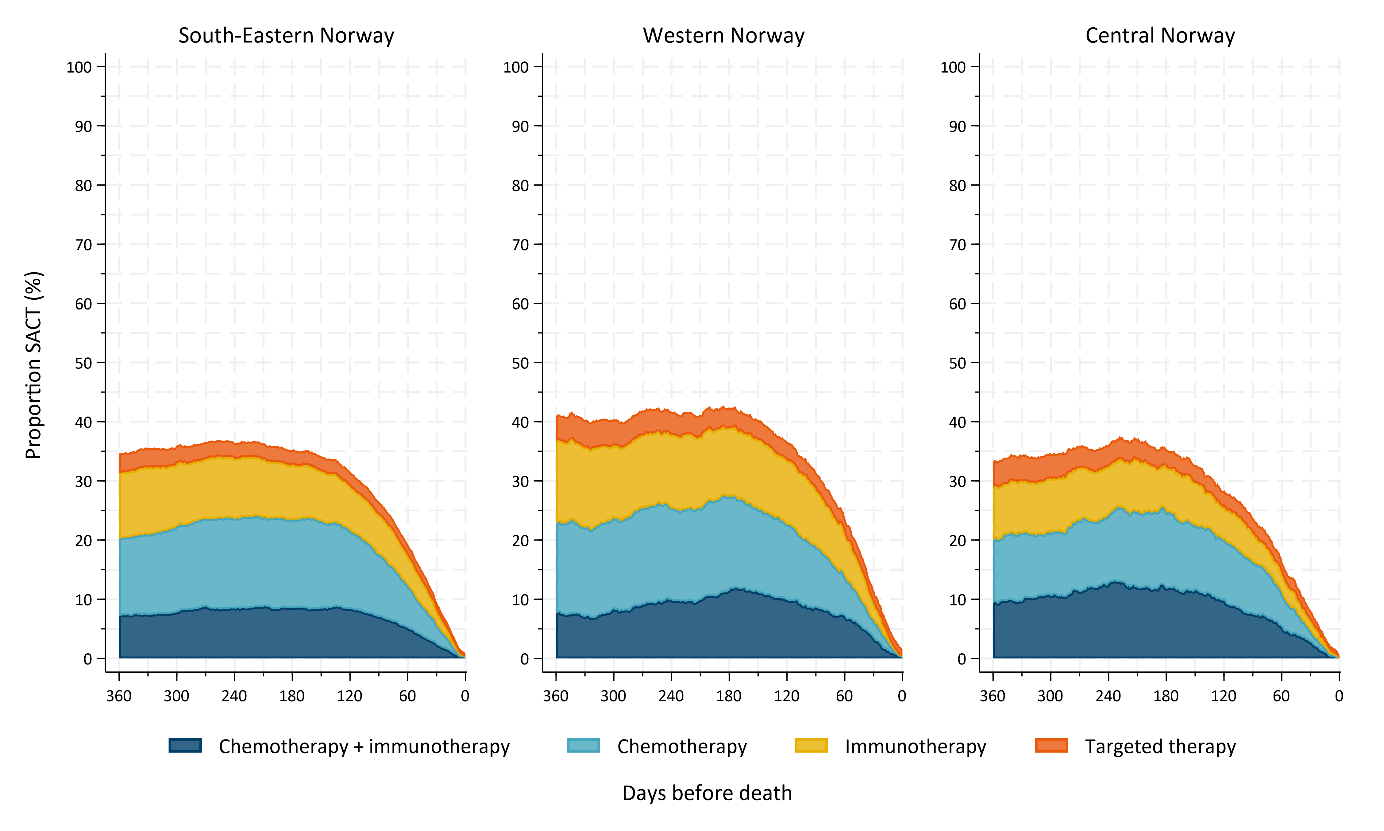
**Figure S6. Lung cancer patients in Norway dying 2020-2023. The proportions of types of SACT by geographical regions.**


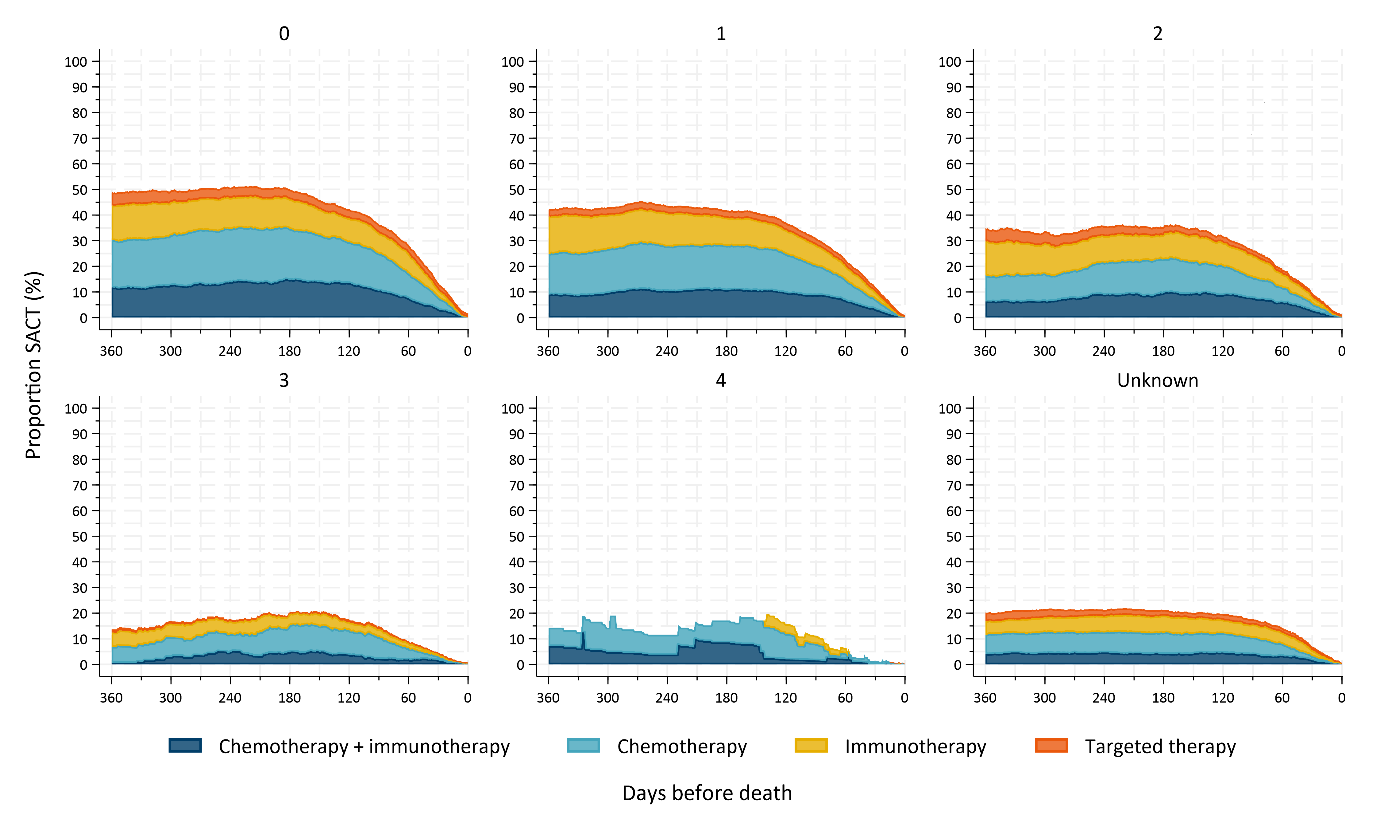
**Figure S7. Lung cancer patients in Norway dying 2020-2023. The proportions of types of SACT by ECOG PS.**
